# Drug-selected population in melanoma A2058 cells as melanoma stem-like cells retained angiogenic features – the potential roles of heparan-sulfate binding ANGPTL4 protein

**DOI:** 10.18632/aging.103890

**Published:** 2020-11-10

**Authors:** Chia-Yu Shih, Yu-Che Cheng, ChiaoHui Hsieh, TingTing Tseng, ShihSheng Jiang, Shao-Chen Lee

**Affiliations:** 1 School of Medicine, College of Medicine, Fu Jen Catholic University, New Taipei, Taiwan; 2 Proteomics Laboratory, Cathay Medical Research Institute, Cathay General Hospital, Taipei, Taiwan; 3 Department of Biomedical Science and Engineering, National Central University, Jhongli, Taiwan; 4 National Institute of Cancer Research, National Health Research Institutes, Miaoli, Taiwan

**Keywords:** drug resistance, melanoma stem-like cells, angiopoietin-like 4 protein

## Abstract

Malignant cancer may contain highly heterogeneous populations of cells, including stem-like cells which were resistant to chemotherapy agents, radiation, mechanical stress, and immune surveillance. The characterization of these specific subpopulations might be critical to develop novel strategy to remove malignant tumors.

We selected and enriched small population of human melanoma A2058 cells by repetitive selection cycles (selection, restoration, and amplification). These subpopulation of melanoma cells persisted the characteristics of slower cell proliferation, enhanced drug-resistance, elevated percentage of side population as analyzed by Hoechst33342 exclusion, *in vitro* sphere formation, and *in vivo* xenograft tumor formation by small amount of tumor cells. The selected populations would be melanoma stem-like cells with high expression of stem cell markers and altered kinase activation. Microarray and bioinformatics analysis highlighted the high expression of angiopoietin-like 4 protein in drug-selected melanoma stem-like cells. Further validation by specific shRNA demonstrated the role of angiopoietin-like 4 protein in drug-selected subpopulation associated with enhanced drug-resistance, sphere formation, reduced kinase activation, *in vitro* tube-forming ability correlated with heparan-sulfate proteoglycans.

Our finding would be applicable to explore the mechanism of melanoma stemness and use angiopoietin-like 4 as potential biomarkers to identify melanoma stem-like cells.

## INTRODUCTION

Melanoma is the highly aggressive skin cancer with high morbidity, high mortality, and poor prognosis. Surgical removal of melanoma *in situ* is the major management [1], while it is difficult to remove completely once re-occurrence with distant metastasis may happen [2]. Cancer cells are highly gene-mutated, heterogeneous, and more-resistant to chemicals, mechanical stress, and immune surveillance. Tumor heterogeneity arises from subpopulations of tumor cells with distinct molecular and biological phenotypes. Different subpopulations would be intrinsically generated by differentiation of cancer stem cells (CSCs) [3, 4] or acquired selection of mutation upon drug treatments [5, 6].

CSCs were recognized as tumor-initiating cells with the characteristics of self-renewal, cell quiescence, and drug resistance, by which derived by altered gene expression, altered cell signaling, or change in epithelial-mesenchymal programming [7–10]. Clinical elimination of bulk tumor might relief tumor malignancy in short term but may relapse after long-term period. Many literatures suggested varieties of specific markers identified in melanoma cells to explain their cancer stemness, drug-resistance, and malignancy [7, 8, 11]. Since cancer therapies are to remove sensitive tumor cells while resistant cells remained survived, whether drug-selected subpopulation were presented as cancer stem-like cells remained of debut.

In this paper, we selected drug-resistant population from melanoma cell lines by repeated cycles of treatments, and compared their phenotypes and genotypes with parental cells. We identified several melanoma- stem-like markers were identified as well one new potential target, angiopoietin-like protein4 (ANGPTL4), was highly expressed in drug-selected subpopulation. Suppression of ANGPTL4 expression by specific shRNA further validated its roles in several cellular activities and phenotypes. This strategy and analysis of these drug-selected subpopulations would be useful to discover new diagnostic markers or targeting mechanisms.

## RESULTS

### Selection and characterization of drug-selected subpopulation in melanoma cells

**Figure 1:**
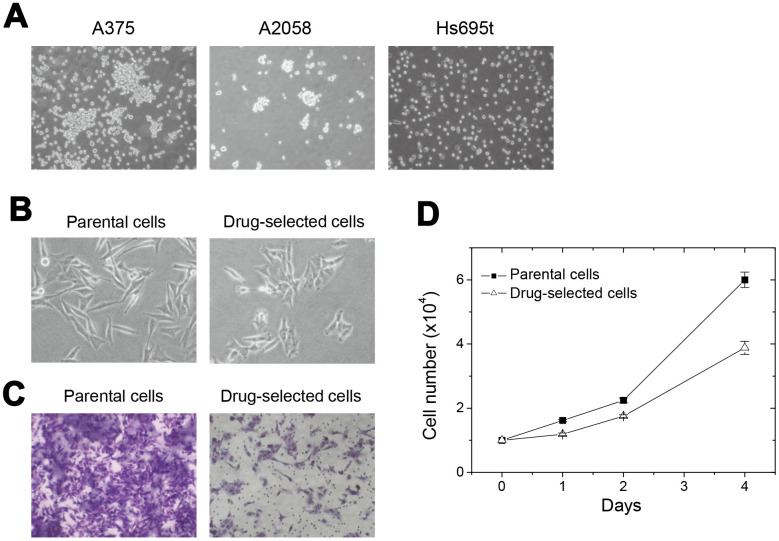
**Selected drug-resistant cells from melanoma A2058 cells showed reduced cell invasiveness and cell proliferation.** (**A**) appearance of cell aggregates or cell spheres under detachment-impaired suspension cultures of different melanoma cells. (**B**) Difference in the phenotypes of elongated parental and pyramid-shaped drug-selected cells. (**C**) Comparison of transwell cell migration ability between parental and drug-selected cells show low cell invasiveness in drug-selected cells. (**D**) cell proliferation was slower in drug-selected cells than in parental cells.

We cultured different melanoma cells under detachment-impaired dishes, which enabled suspension culture and cell sphere formation. As seen in Figure 1A, most of the suspended melanoma cells formed irregular aggregates except Hs695t cells. For melanoma A375 cells, suspended cells accumulated but cell boundary remained distinguishable. Melanoma A2058 cells could partially form larger cell spheres, which implied the presence of cancer stem-like cells. It was known that stem-like subpopulation of tumor cells would be drug-resistant [12–14], so that we tried to enrich them by drug selection.

We treated melanoma A2058 cells using either one of different therapeutic agents with partial response in clinical trials. Sorafenib is the multikinase inhibitor that had been used to inhibit tumor cell proliferation. Sorafenib has been evaluated as a single therapy agent as well in combination with various chemotherapeutical drugs in several clinical trials [15, 16]. Carmustine is one of alkylation agents to interfere DNA replication and RNA transcription. It had been included as one component in Dartmouth regimen (carmustine, cisplatin, dacarbazine, and tamoxifen) in melanoma therapies [17–19]. Upon treatment with 20 μM sorafenib for 3 days, the cell proliferation was reduced as observed in significantly low cell numbers. Treatment with 20 μM carmustine resulted in significant cell death as seen with detached cells. Further suspension culture of A2058 cells after sorafenib treatment didn’t exhibit the characteristic cell spheres, while those after carmustine treatment retained the formation of cell sphere (data not shown).

To enrich these drug-selected subpopulation of melanoma A2058 cells, we sequentially and repeatedly selected by several cycles of carmustine treatments. Repeated cycles of selection, restoration, and cell amplification were done for several months, and the concentrations of carmustine used for selection were gradually increased from 20 μM to 100 μM. The drug-selected cells were less elongated (Figure 1B) and the invasion ability were largely-reduced than parental cells (Figure 1C). It suggested that these drug-selected cells were low-invasive. As seen in Figure 1D, drug-selected cells proliferated slower comparing with parental melanoma cells. Slower proliferation and less invasive ability might suggest the drug-selected subpopulations were less-malignant in cell studies. Since we suspected the selected subpopulation was melanoma-stem like cells, further characterization at the ability of sphere formation, content of side population, and xenograft tumor formation were performed.

**Figure 2:**
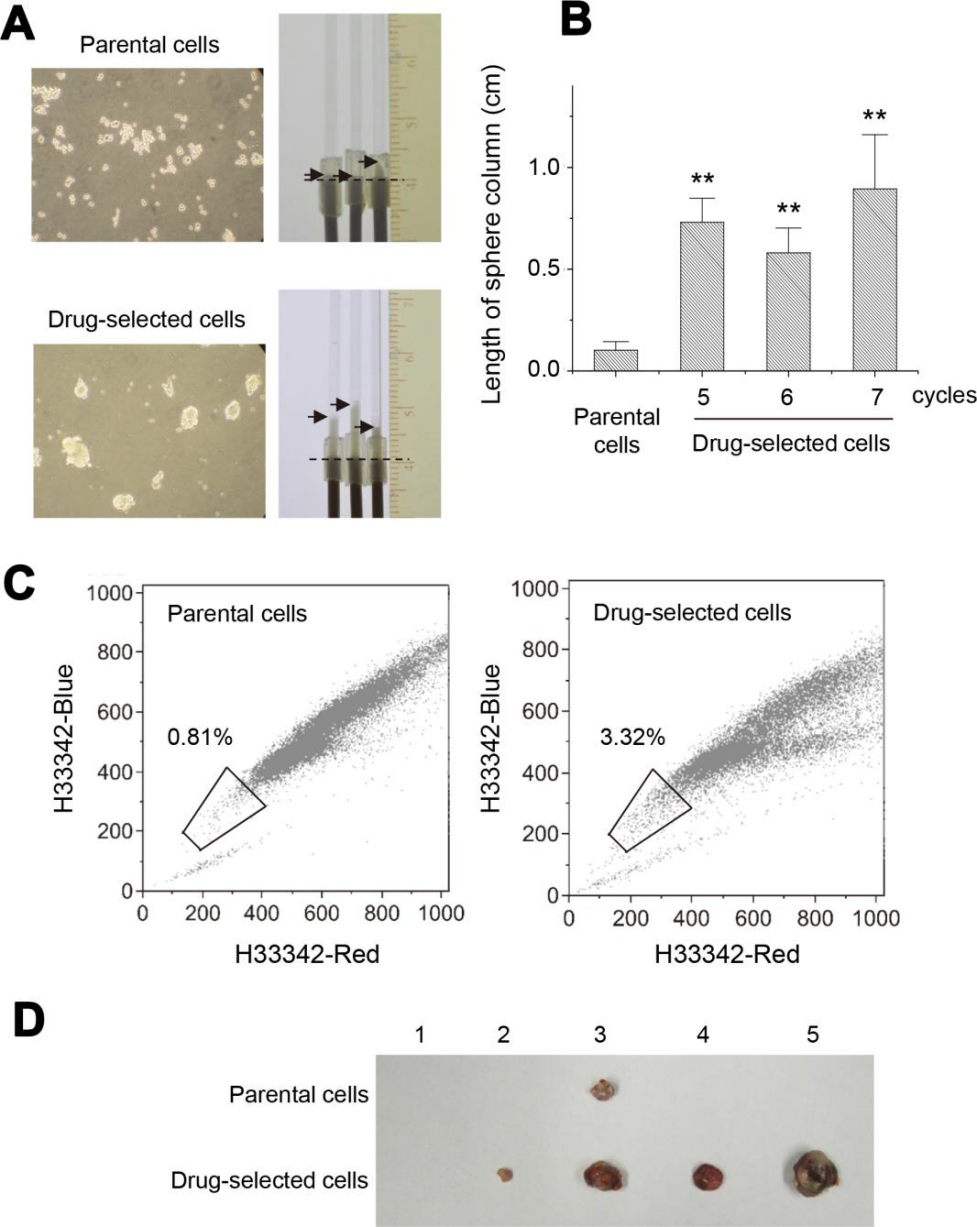
**Drug-selected melanoma cells persisted the characteristics of melanoma-stem like cells.** (**A**) cell sphere forming assay under suspension culture for 2 weeks. Cell spheres were formed by drug-selected cells, while parental cells formed mostly cell aggregates. Spherocrit assay showed longer sphere length in capillary tube by cell spheres from drug-selected cells. (**B**) Quantification of sphere lengths from drug-selected cells through different selection cycles. (**C**) Side population analysis of parental and drug-selected cells showed more percentage of side population cells as determined by low dual fluorescence of hoechst33342-red and hoechst33342-blue. (**D**) Animal experiments of subcutaneous tumor formation showed larger tumors derived from drug-selected cells.

*In vitro* sphere formation was used to demonstrate the activity of cancer stem cells. As seen in Figure 2A, suspension culture of parental cells mostly generated cell aggregates but those of drug-selected cells produced cell spheres. We had developed one novel approach, “spherocrit assay”, to quantify the level of sphere formation [20]. The number and the size of cell spheres both reflected their sphere forming abilities. Rigid and compacted spheres were collected into microcapillary tubes. The length of sphere column reflected the sphere forming ability. As seen in Figure 2A, drug-selected cells had longer sphere columns than parental cells did. Comparing the sphere column length generated from drug-selected cells after selection by 5, 6, or 7 cycles, they all had similar sphere-forming ability and were higher than that of parental A2058 cells (Figure 2B). This indicated that the period of long-term and repeated selection for 5 months was sufficient to enrich melanoma stem-like cells.

Next, cell fractions of side population were analyzed by Hoechst33342-exclusion assay on flow cytometer (Figure 2C). The content of side population in drug-selected cells was 3.32 % comparing with 0.81% in parental melanoma cells, which reinforced the enrichment of cancer-stem like cells after repeated selections. In addition, critical feature of cancer stem cells was the ability to form tumors by small number of cells. We performed xenograft tumor formation experiments in nude mice by inoculation of 2 x 10^3^ cells subcutaneously. As shown in Figure 2D, 4 out of 5 mice developed subcutaneous tumors within 3 months, and drug-selected cells generated larger tumors than parental cells did. Those data indicated this drug-selected subpopulation of melanoma cells were potential melanoma stem-like cells, which had features of slower proliferation, less cell invasion, higher sphere-forming ability, more side population cells, and higher tumor malignancy *in vivo*.

### Melanoma-stem marker expression and altered protein phosphorylation in drug-selected melanoma cells

**Figure 3:**
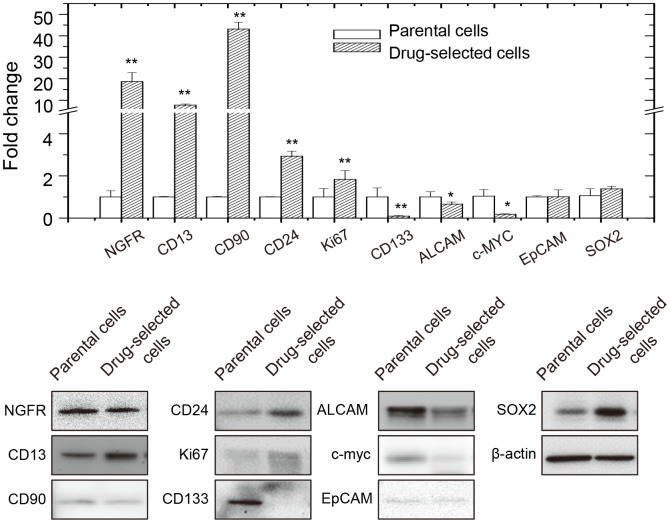
**Expression of melanoma-stem markers in parental or drug-selected cells as analyzed by qPCR and western blot.**

We also analyzed the expression of known melanoma-stem markers and potential signaling pathways. As seen in Figure 3, expression levels of several melanoma stem cell markers (*NGFR, NES, ABCB5, BMI1, CXCR6, CD13, CD90, CD133, CD44, ABCG2, EpCAM*, and *CD20* [21–25]) were analyzed in parental or drug-selected cells. Of them, mRNA expression levels of some markers were higher but other markers were lower in drug-selected subpopulations than in those of parental cells as determined by qPCR. Further evaluation by western blot analysis also confirmed the increased expression of CD13, CD24, Ki67, and Sox2, but decreased CD133, ALCAM, and c-myc in drug-selected subpopulation (Figure 3). This suggested the melanoma stem-like characteristics in drug-selected cells as CD13^+^CD24^+^.

**Figure 4:**
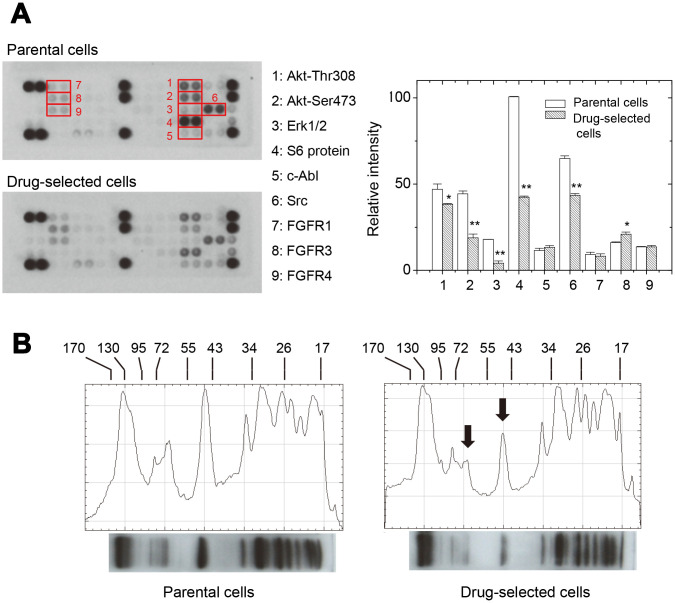
**Drug-selected melanoma cells had lower levels of kinase activation than parental cells.** (**A**) phosphor-kinase antibody array showed differential levels of different phosphor-proteins in parental or drug-selected cells. The quantification of blot intensities were shown (n=2; data were mean ±SD; *, *p* < 0.05.). (**B**) Western blot analysis of phosphor-tyrosine proteins in parental or drug-selected cells. Significantly lower levels of phosphor-proteins in drug-selected cells were indicated by arrows. Line-histograms were generated by Image J.

In addition, we analyzed the pathway activations for different receptor tyrosine kinases using antibody array. As seen in Figure 4A, the levels of several kinase phosphorylation were different between parental and drug-selected cells according to the dot intensities shown on the blot membranes. Of them, the phosphorylation of Akt-Thr308, Akt-Ser473, Erk1/2, S6 kinase, and Src were less in drug-selected cells. Only the phosphorylation of fibroblast growth factor receptor 3 (FGFR3) was higher in drug-selected cells.

The expression patterns of phosphor-tyrosine proteins were also compared by western blot. As seen in Figure 4B, the significant decreases in peak heights for drug- selected cells were seen at those around 44 and 60 kDa (indcaited by arrows). The peak at 44kDa corresponded to the phosphor-ERK (42/44 kDa). The peak at 60 kDa corresponded to the phosphoAkt (60kDa) or phosphor-Src (60kDa). These were consistant with the results from antibody array experiments (Figure 4A). The decrease in phospho-S6kinase (70/85 kDa) and increase in phospho-FGFR3 (125~165 kDa) were not significantly observed.

### Molecular profiling of drug-selected melanoma cells showed high expression of ANGPTL4

In order to evaluate the mechanism why drug-selected cells were more malignant but the cell proliferation/cell invasion ability was less malignant, the gene expression profiling in parental and drug-selected melanoma cells were done using cDNA IlluminaBbeAdarray. Within all the 47,323 probes, there were 2,948 targets (2,445 targets upregulated; 503 targets downregulated) got changed with statistical significance. The microarray results were deposited at Gene Expression Omnibus with accession number GSE135689. These targets were analyzed by DAVID (Database for Annotation, Visualization and Integrated Discovery) v6.7 for functional gene clustering in different gene ontologies (biological process, cellular component, and molecular function). The biological processes perturbed by selection of drug-resistance were associated with angiogenesis, cell migration, survival, cell adhesion, which account for tumor malignancy. Stimulus responding, inflammation, and morphogenesis might associate with the feature of cancer stemness. In addition, almost all the changed genes were located at extracellular or plasma membrane regions, which explained the aforementioned change in biological processes. As to the aspects of molecular functions, they implied the receptor dimerization, as well as the glycosaminoglycan-binding, and the following signaling events would be dominant in drug-selected cells.

By the way, in the results of microarray profiling, NGFR, CD13, CD90, EpCAM, and Ki67 were significantly increased in drug-selected cell, while those of CD133 was downregulated. These were consistent with the aforementioned qPCR analysis (Figure 3). Several ABC family proteins, such as ABCA8, ABCC3, ABCA1, ABCG1, and ABCA9, were upregulated in drug- selected cells as seen in microarray data. As to epithelial-mesenchymal transition proteins, the expression levels of vimentin, E-cadherin, and N-cadherin were not significantly changed.

**Figure 5:**
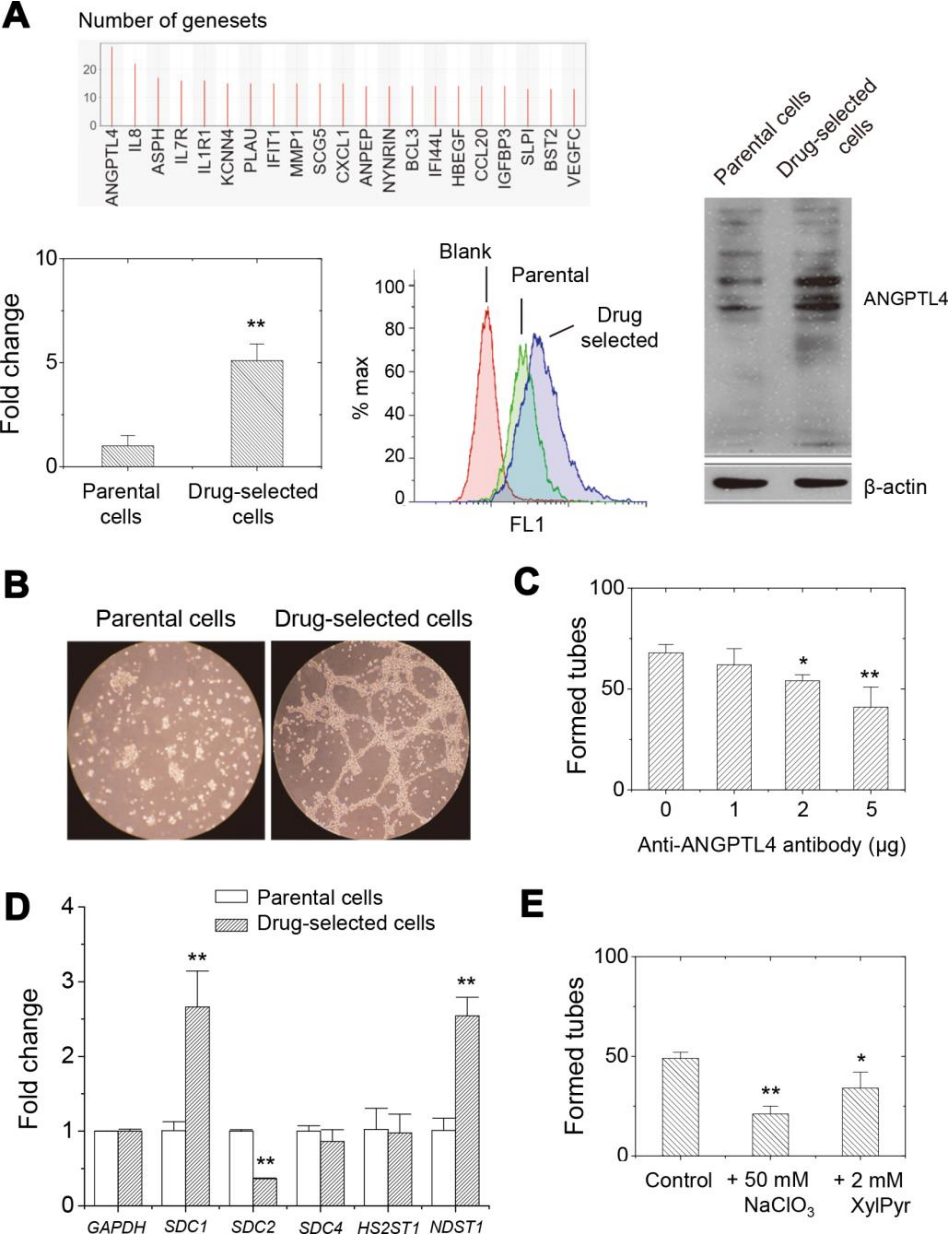
**Higher expression of ANGPTL4 in drug-selected cells contributed to angiogenic activity through glycosaminoglycan modification on proteoglycans.** (**A**) GSEA analysis showed that ANGPTL4 was the enriched in the gene sets related to oncogenic features. qPCR and western blot analysis demonstrated the higher expression level of *ANGPTL4* in drug-selected cells. (**B**) Tube formation assay showed significantly connected and aligned cells by drug-selected cells, but not seen by parental cells. (**C**) Pretreatment with anti-ANGPTL4 antibody reduced tube-forming activity of drug-selected cells. (n=4; data were mean ±SD; **, *p* < 0.01; *, *p* < 0.05.) (**D**) qPCR analysis at expression of several heparan-sulfate proteoglycans (*SDC1*, *SDC2*, and *SDC4*) and sulfate transferases (*HS2ST1* and *NDST1*) in parental and drug-selected cells. (**E**) Pretreatment with chemical inhibitors to reduce sulfate groups (by NaClO_3_, sodium chlorate) or carbohydrate chain biosynthesis (by XylPyr, xylopyroside) inhibited tube-forming activity of drug-selected cells. (n=4; data were mean ±SD; **, *p* < 0.01; *, *p* < 0.05.).

In order to specify the potential targets, we performed GSEA (gene set enrichment analysis) and leading edge analysis in the microarray results. It showed 187 upregulated and 1 downregulated gene sets out of 188 oncogenic signature gene sets (C6 collection in MSigDB v4.0) with significantly enrichment at nominal *p*-value < 1% upon drug-resistance. The leading edge analysis revealed *ANGPTL4* and *IL8* gene were mostly potential associated with oncogenic features upon selection of drug-resistance (Figure 5A). The microarray results also indicated ~50-folded and ~500-folded increased in *ANGPTL4* and *IL8* expression, respectively. The qPCR analysis further confirmed the increased expression in *ANGPTL4* and *IL8* gene expression in drug- selected cells.

It was known the increased expression of IL-8 protein correlated with melanoma malignancy. IL-8 protein in melanoma cells was recognized as autocrine [26] and its expression level was correlated with metastatic potential [27, 28]. IL-8 would stimulate multiple cell signaling pathways, including PI3K-AKT/PKC/MEK-ERK pathways in melanoma cells [29]. However, the ERK phosphorylation in drug-selected cells was decreased. The ANGPTL4 proteins belong to a superfamily of angiogenic proteins involved in lipid metabolism, energy homoeostasis, inflammation, cell differentiation, angiogenesis, or tumorigenesis, [30]. Previous literature indicated the expression of ANGPTL4 led to reduced ERK phosphorylation which was mediated by *C*-terminal domain [31]. In our selected subpopulation with high ANGPTL4 expression, we did see reduced ERK phosphorylation comparing with that of parental melanoma cells (Figure 4A and 4B). These could explain why drug-selected cells had higher tumorigenic potential but lower ERK phosphorylation. The role of ANGPTL4 protein in drug-selected melanoma cells might be important.

### Upregulation of ANGPTL4 in drug-selected cells enabled their tube formation *in vitro* through sulfated proteoglycan

The expression level of *ANGPTL4* was validated in parental or drug-selected cells. As seen in Figure 5A, qPCR analysis showed 5-folded increase in mRNA level for drug-selected cells. Cell surface expression level was higher in drug-selected cells as examined by flow cytometry (Figure 5A). Western blot showed increase ANGPTL4 protein bands at 45 and 54 kDa (glycosylated form), which confirmed the increased expression of *ANGPTL4* gene. Another results from ELISA assay (data not shown here) also showed the higher secretion of ANGPTL4 protein which was consistent with the previous finding.

Since ANGPTL4 was known as angiogenic protein, we examined the *in vitro* angiogenic activity of parental and drug-selected cells by tube formation assay. As shown in Figure 5B, no *in vitro* tube was formed by parental cells. However, significant tubes were generated by drug- selected cells. In order to confirm that *in vitro* tube-forming was associated with ANGPTL4 protein secreted by drug-selected cells, we pretreated the cells with anti-ANGPTL4 antibody. As seen in Figure 5C, the angiogenic activity was indeed inhibited by anti-ANGPTL4 antibody which implied the *in vitro* tube formation was contributed by secreted ANGPTL4. In addition to higher expression of ANGPTL4 protein contributing higher angiogenic ability, we suspected some membrane receptor might also be responsible and was upregulated in drug-selected cells.

ANGPTL4 was previously recognized as potential glycosaminoglycan-binding proteins with preference toward heparan sulfate [32, 33]. We analyzed the expression level of syndecan-1 (SDC1), syndecan-2, and syndecan-4, as well as the biosynthetic enzymes associated with the levels of *N*-sulfate and 2-*O*-sulfate groups, in parental and drug-selected cells. *N*-sulfate was introduced by the enzymes of *N*-deacetylase/*N*-sulfotransferase (*NDST* gene products), which contains 4 different members. Addition of 2-*O*-sulfate group was achieved by solely heparan sulfate 2-*O*-sulfotransferase 1 (*HS2ST1* gene product). In melanoma A2058 cells, only the expression of *NDST1* gene was detected (data not shown). We analyzed the expression levels of these genes by qPCR. As seen in Figure 5D, *SDC1* expression was higher (~2.5-folded) but *SDC2* expression was less (~0.3-folded) in drug-selected cells. There was no significant difference in *SDC4* expression between parental and drug-selected cells. The *HS2ST1* gene expression was similar between parental and stem-like melanoma cells, but *NDST1* gene expression was elevated at stem-like melanoma cells comparing with parental melanoma cells.

Experiment using biosynthesis inhibitors further conformed the importance of heparan sulfate in angiogenic ability of drug-selected cells. Sodium chlorate was used to abolish sulfate groups on glycosaminoglycan chains; while xylopyranoside was used to inhibit biosynthesis of glycosaminoglycan chain. As seen in Figure 5E, both sodium chlorate or xylopyranoside suppressed the level of in vitro tube formation. These results suggested membrane proteoglycan SDC1 and *N*-deacetylase/*N*-sulfotransferase-1 (NDST1) might associate with melanoma stemness, and its heparan sulfate chain might be the acceptors for ANGPTL4.

### ANGPTL4 accounted for partial features of drug-selected subpopulation

**Figure 6:**
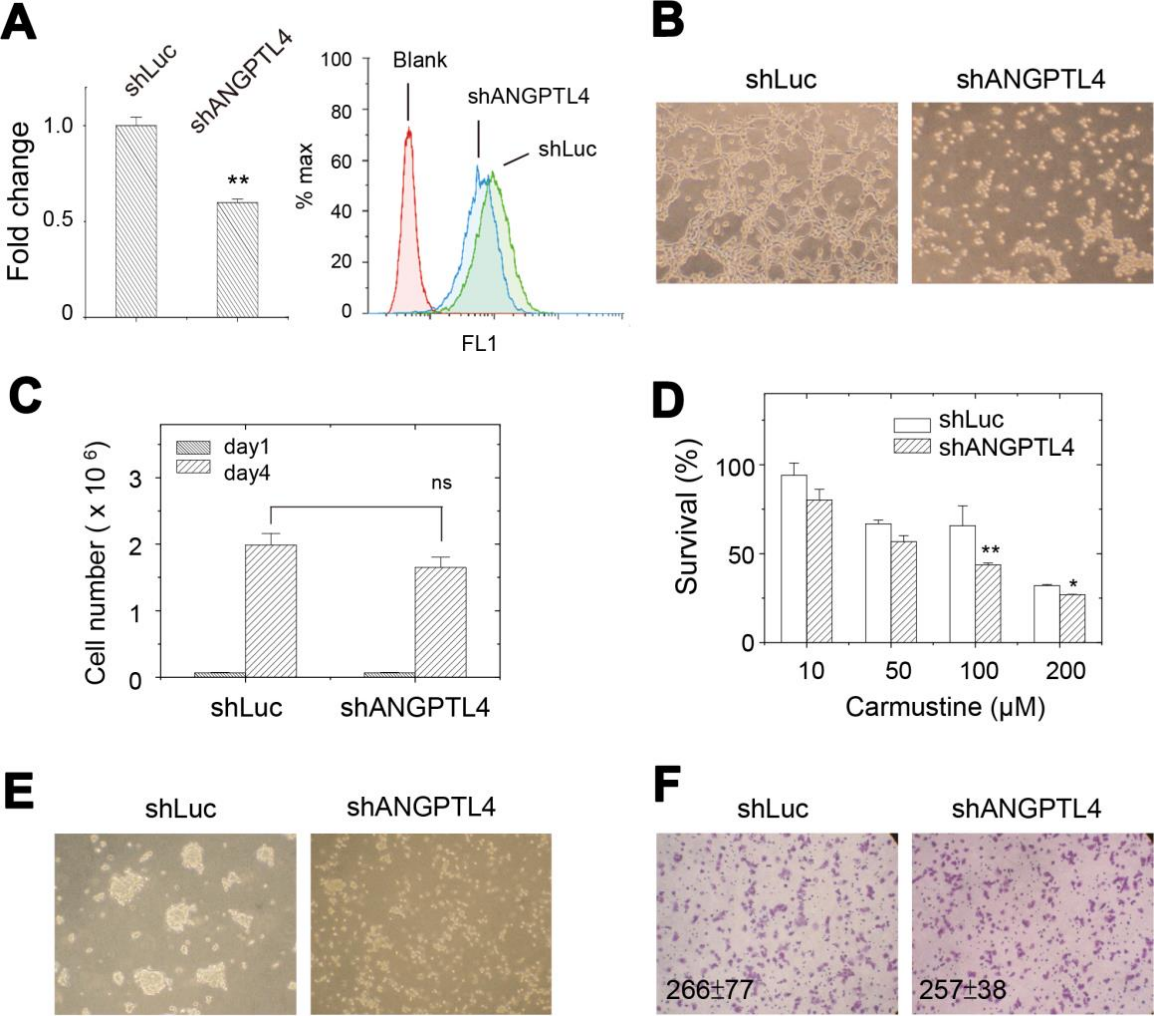
**Effects of suppression of ANGPTL4 expression on cellular activities in drug-selected cells.** (**A**) ANGPTL4 expression level was reduced by transfection of ANGPTL4-specific shRNA as examined by qPCR and flow cytometry. (n=3; data were mean ±SD; **, *p* < 0.01) (**B**) *in vitro* tube forming ability of drug-selected cells was abolished by the suppression of ANGPTL4 expression. (**C**) cell proliferation assay showed no difference in cell number between parental and drug-selected cells. (n=3; data were mean ±SD; *ns*: not significant with *p* = 0.08). (**D**) upon suppression of ANGPTL4 expression in drug-selected cells, the sensitivity toward carmustine was increased as indicated by less survival. (n=6; data were mean ±SD; **, *p* < 0.01; *, *p* < 0.05.). (**E**) sphere forming ability was abolished by suppression of ANGPTL4 expression. (**F**) transwell assay showed no difference in migrated cells between parental and drug-selected cells. (n=6; data were mean ±SD).

Since high expression of ANGPTL4 was observed in drug-selected cells, we further investigated the roles of ANGPTL4 in cellular activities of drug-selected cell population. We suppressed the ANGPTL4 expression by transfection of specific shRNA against ANGPTL4 (shANGPTL4). Upon shANGPTL4 transfection, the mRNA expression and surface expression levels were all decreased as checked by qPCR and flow cytometry, respectively (Figure 6A). Since ANGPTL4 protein was known as angiogenetic factor and its higher expression correlated with tube forming ability (Figure 5B), we examined the effect of shANGPTL4 transfection on tube-forming ability of drug-selected cells. As shown in Figure 6B, suppression of ANGPTL4 expression did abolish tube formation, which reinforced the role of upregulated ANGPTL4 in promoting tumor malignancy.

Upon the suppression of ANGPTL4 expression, cell proliferation was slightly reduced (see Figure 6C, *p*-value > 0.05). It suggested that ANGPTL4 protein didn’t associate with the feature of slow proliferation in drug-selected cells. In addition, the shANGPTL4-transfected cells were more sensitive to carmustine as seen by less percentage of cell survival (Figure 6D). The effects of shANGPTL4 transfection on different cell activities was also examined. Sphere forming ability was significantly reduced upon shANGPTL4 transfection (Figure 6E). As seen in Figure 6E, the transwell migration ability of drug-selected melanoma cells remained less invasive as seen in Figure 1C. These suggested that ANGPTL4 protein determined the cell activities of tube formation, sphere formation, and drug-sensitivity.

**Figure 7:**
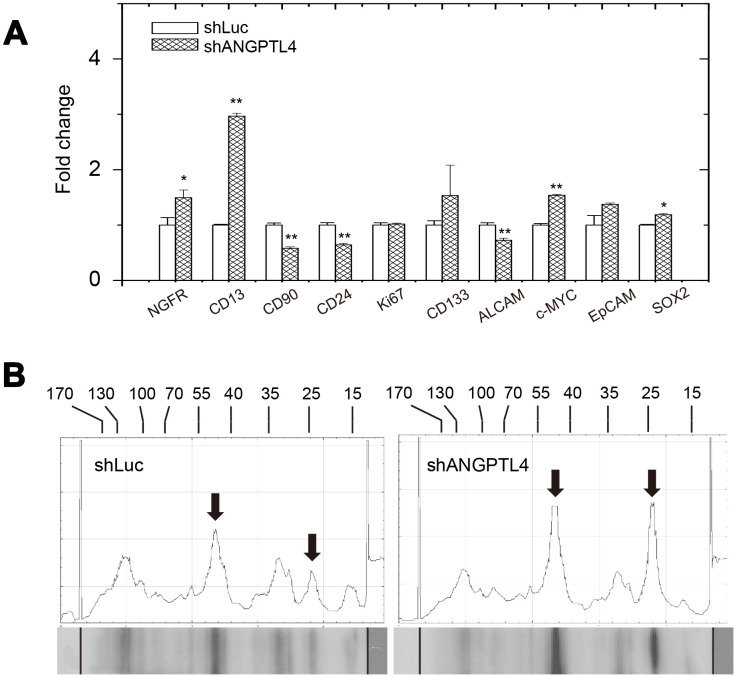
**Effects of suppression of ANGPTL4 expression on melanoma-stem marker expression and phosphor-protein expressions in drug-selected cells.** (**A**) Effect of the ANGPTL4 suppression on expression of melanoma-stem markers in drug-selected cells as analyzed by qPCR. (**B**) Effect of the ANGPTL4 suppression on expression of phosphor-tyrosine proteins in drug-selected cells as analyzed by western blot analysis. Significantly higher levels of phosphor-proteins in drug-selected cells were indicated by arrows. Line-histograms were generated by Image J.

We also examined the expression of melanoma stem-cell markers after suppression of ANGPTL4 expression. As shown in Figure 7A, reduced ANGPTL4 expression led to higher expression of NGFR, CD13, c-myc, SOX2, and lower expression of CD90, CD24, and ALCAM.

Comparing the results of melanoma stem-cell marker expressions in Figure 3 and Figure 7A, we suggested that high expression of ANGPTL4 in melanoma cells might associate with the CD90^+^/CD24^+^ phenotype. The expression patterns of phosphor-tyrosine proteins upon suppression of ANGPTL4 expression was investigated. As seen in Figure 7B, levels of phosphor-proteins with the molecular sizes of 44 and 24 kDa were increased by the suppression of ANGPTL4 expression. This suggested that reduced level of phospho-ERK in drug-selected melanoma cells was mediated by higher expression of ANGPTL4, which is consistent with previous observation [31]. The level of phosphor-protein of 24 kDa was not significantly different in drug-selected melanoma cells comparing that in parental melanoma cells. In summary, we suggested the higher ANGPTL4 expression in drug-selected melanoma cell subpopulation account for melanoma-stemness features of *in vitro* tube formation ability, sphere formation, drug-resistance, and reduced ERK phosphorylation.

## DISCUSSION

The failure of tumor therapy was from drug resistance of tumor cells, which composed of different subpopulations with different sensitivities toward chemoagents. The source of tumor heterogeneity might be explained by two different models [34, 35]. Colonal evolution model suggests the presence of equipotent cancer cells with different phenotypes and different proliferation potentials. Stochastic stimulation caused mutation and evolved into different cell subpopulations with different drug sensitivities. Another well-known model, CSCs model, suggests the presence of clonogenic cells to generate different types of tumor cells through unusual differentiation potential that enabled tumor heterogeneity with different drug sensitivities. Resembling stem cells, CSCs are small population of cells with stem cell-like ability, which include: high self-renewal capacity, asymmetric division into heterogeneous lineages, extremely resistant to drug or stress, and sustained growth. Most studies aimed to discover CSC-specific markers for the purpose of clinical diagnosis or treat as therapeutic targets. Nevertheless, tumor evolution after drug-treatment might depend on each individual, but the outcome was the resistance toward tumor therapy, metastasis, and poor prognosis with relapse.

In our current study, we focused at the strategy to address the co-features of drug-resistance, tumor malignancy, and melanoma stemness. The drug-selected cells were more resistant to the treatment of carmustine or paclitaxel (Supplementary Figure 1). We also characterized potential new marker that might associate with tumor malignancy. We demonstrated the drug-selected subpopulations had high tumor-initiating potential as seen by elevated xenograft tumor growth (Figure 2D). They also had common features of *in vitro* sphere-forming ability (Figure 2A and 2B), higher percentage of side-population (Figure 2C), and higher expression of known melanoma-stem markers (Figure 3).

Signaling mechanisms may be dysregulated in cancer cells to allow uncontrolled self-renewal in either stem cells or progenitor cells [36]. The self-renewal of CSCs associated with asymmetric cell division is one of the hypotheses to explain tumor heterogeneity [36, 37]. It can be guided by the activation of several pathways, including Wnt, Notch, Hedgehog, and others [37]. A CSC may autonomously trigger the appropriate signaling cascade to maintain self-renewal with minimal niche support. In this paper, we demonstrated the significantly deactivated ERK and Akt pathways in drug-selected cells. This was consistent with the facts at slower cell proliferation (Figure 1D) and less cell invasiveness (Figure 1C), that implied the dominant feature of CSCs. However, higher tumor-initiating ability was seen in drug-selected cells by elevated xenograft tumor growth (Figure 2D). Slower cell proliferation but larger xenograft tumor formation by drug-selected cells was observed. We explained that slower cell proliferation would be driven by decreased levels of tyrosine phosphorylation (Figure 4), but the larger tumor formation would be the consequence of higher Ki67 level associated with increased IL-8 level.

According to our observation, AKT phosphorylation was less in drug-selected cells where the ANGPTL4 expression were higher. Previous paper suggested that c-terminal domain of ANGPTL4 blocked bFGF-induced ERK activation, but not Akt activation, in HUVEC cells [31]. We thought that ANGPTL4 protein might block the effect of tyrosine receptor kinase through competition with growth factors for binding with heparin sulfate proteoglycans. This issue needs more investigation in the future. However, it is likely that CSCs might need appropriate microenvironment to provide the stimuli for their self-renewal. The interaction of CSCs with their microenvironment or acceptance of extracellular stimulus will be of great importance to modulate their activities.

We identified ANGPTL4 protein was highly expressed in drug-selected subpopulation. ANGPTL4 is ubiquitously expressed at liver, adipose tissue, blood plasma, placenta, intestine and heart tissue [38]. ANGPTL4 was originally referred as fasting-induced adipose factor, which was an important regulator of lipoprotein lipase activity [38, 39]. ANGPTL4 involved in fatty acid-driven feedback mechanism that was regulated by fatty acids through fatty acid-activated peroxisome proliferator activated receptors [40]. LPL hydrolyzed triglycerides thus reduced the levels of circulating triglycerides. Therefore, ANGPTL4 inhibited LPL activity that promoted uptakes of plasma triglycerides at exercising muscle and brown adipocytes. Not only involved in lipid metabolism, ANGPTL4 also involved in energy homoeostasis, inflammation, wound healing, cell differentiation, angiogenesis, tumorigenesis, and pathogenesis of nephrotic symptom [30]. It was also induced by hypoxia through hypoxia-induced factor 1α [40]. *ANGPTL4* mRNA has been found to be upregulated in the perinecrotic areas of many human tumors, renal carcinomas, squamous cell carcinomas, and gastric cancers [41]. Its expression level increased as tumors progressed from benign to metastatic states [42]. Tumor-derived ANGPTL4 conferred anoikis resistance of tumor cells via autocrine adhesion mimicry [42]. It interacted with β1 and β5 integrins, thus hijacking integrin-mediated signaling to maintain an elevated oncogenic O_2_^−^ /H_2_O_2_ ratio. This stimulates the redox-mediated activation of the Src machinery and activates the downstream PI3K/Akt and ERK signaling cascade to promote cell survival and tumor growth [42]. ANGPTL4 knockdown enhanced cell apoptosis and sensitized tumor cells to drug treatment, confirming that ANGPTL4 played a key role in anoikis resistance.

The role of ANGPTL4 in angiogenesis and vascular leakiness remains controversial. Some studies proposed the anti-angiogenic role of ANGPTL4 that it could prevent metastasis through inhibition of angiogenesis, tumor cell motility and invasion [32, 43]. However, other reports proposed that ANGPTL4 protein was pro-angiogenic and pro-metastatic. ANGPTL4 protein significantly promoted *in vitro* sprouting of vascular endothelial cells [44]. *ANGPTL4* had been identified as one of the genes predicting breast cancer to lung metastasis with the greatest frequency [45]. It was further demonstrated that TGFβ-induced ANGPTL4 enhances the retention of cancer cells in the lungs, disrupted vascular endothelial cell–cell junctions, increased the permeability of the lung capillaries, and facilitated the trans-endothelial passage of tumor cells, thus promoted the critical steps of metastasis [46]. One literature suggested that *C*-terminal fibrinogen-like domain of ANGPTL4 disrupted the endothelial continuity by interacting with integrin α5β1, VE-cadherin, and claudin-5 thus facilitating metastasis [47]. Nevertheless, these evidences implied ANGPTL4 is potential target in tumor therapy. In this paper, we demonstrated the roles of ANGPTL4 protein would be pro-angiogenic (Figure 5B and 5C). This was consistent with the previous papers about the relationship between cancer stem-like cells and angiogenesis in different types of cancer, including melanoma [48–52]. Several literatures suggested the specific interaction of ANGPTL4 with surface component, heparin sulfate, but not with other glycosaminoglycans [33]. These potentially affected the ANGPTL4 activity in different aspects [32, 33, 53]. In our current study, we demonstrated the specific expression of heparan sulfate proteoglycan (SDC1) and sulfate transferase (NDST1) in drug-selected cells (Figure 5D), and suggested the importance of sulfate groups in angiogenic activity of ANGPTL4 protein (Figure 5E).

In this paper, we showed high expression of several melanoma-stem cell markers (Figure 3). After further investigation using ANGPTL4-specific shRNA to suppress the protein expression, we showed that ANGPTL4 might correlate with the high expression of CD90 and CD24 (Figure 7A). CD24 was shown to be important marker of poor prognosis or marker for tumorigenesis in malignant melanoma [54, 55]. Potentially, CD24 expression was mediated by STAT3-dependent /SOX2-mediated pathways that was correlated with adaptive resistance toward targeted therapy in melanoma [56, 57]. Our study showed CD24 was downregulated by suppression of ANGPTL4 expression in drug-selected cell subpopulation. This might be critical to develop ANGPTL4-targeted or proteoglycan-targeted strategies against adaptive resistance in melanoma therapy.

## MATERIALS AND METHODS

### Cell culture and selection of drug-selected cell populations in melanoma cells

Human melanoma A2058, A375, and Hs695t cells were maintained in culture dish (Corning Incorporated Life Sciences, Tewksbury, MA, USA) supplemented with DMEM medium containing 10 % (v/v) fetal bovine serum, 100 units/mL penicillin, and 100 μg/mL streptomycin at 37°C under 100% humidity.

To enrich carmustine-resistant melanoma cell subpopulation, melanoma A2058 cells were plated at density of 2x10^6^ cells for initial selection. The enrichment was done by repetitive cycles of chemoagent treatment, removal of chemoagent, and cell restoration/proliferation, which would be completed in one month. One enrichment cycle was completed till the optimal growth rate achieved with no cell detachment/death. Initially, melanoma cells were treated by 20 μM carmustine for 3 days and the concentrations of chemoagents used to select drug-resistant cells in each cycle were then gradually increased up to 100 uM. Totally 5 to 7 enrichment cycles were done to obtain drug-selected subpopulation of melanoma cells for the following characterizations. In order to compare in parallel, the parental A2058 cells were cultured and maintained along with enrichment procedure of 5-7 months.

ANGPTL4-specific shRNA (clone ID: TRCN0000056723) and control shRNA (shLuc, clone ID: TRCN0000072249) were purchased from the National RNAi Core Facility located at Institute of Molecular Biology/Genomic Research Center, Academia Sinica, Taiwan. Cells were transfected using TurboFect transfection reagent (Thermo Fisher Scientific Inc., Pittsburgh, Pennsylvania, USA) according to the manufacturer’s instruction. The transfected cells were selected and enriched under growth medium containing 5 μg/mL puromycin after shRNA transfection.

### Cell proliferation, transwell migration assay, and cell survival assay

For cell proliferation assay, melanoma cells were collected by trypsinization and were counted by hemocytometer. The 2 x 10^4^ cells were plated onto 35 mm culture dish. The cells were incubated and grew for different day intervals. The cells were then trypsinized and the cell numbers were counted by hemocytometry.

For transwell migration assay, 1x10^5^ cells (in DMEM medium with 2% (v/v) FBS) were applied onto the culture insert (8 um pore size; SPL life sciences, Co. Ltd., Pocheon-si, Gyeonggi-do, Korea). The bottom wells contained 600 uL culture medium with 10% (v/v) FBS, and were left un-agitated in the cell incubator until observation. The migrated cells were visualized by staining with 0.2% (w/v) crystal violet.

For cell survival assay, 1x10^4^ cells were plated into each well of 96-well plate and incubated overnight. Different concentrations of chemoagents were added and incubated for 1 days in culture incubators. The cell viability was determined by Alamar Blue assay (Thermo Fisher Scientific Inc., Pittsburgh, PA, USA) to evaluate the mitochondria activity.

**Sphere formation and spherocrit assay**

For sphere formation, 1 x 10^5^ melanoma cells were seeded in 10-cm sterile plastic dish (Alpha-plus, Inc., TaoYuan, Taiwan) supplemented with 10 mL culture medium. The cell sphere were formed and enlarged for 2 weeks with medium renewal every 2-3 days.

One novel “spherocrit” assay was used to quantify the contents of cell spheres [20]. Cell aggregates or cell spheres cultured for 2-weeks were collected and processed as followed. In general, the cell spheres (rigid-packed cells) or cell aggregates (loose-packed cells) were pipetted to disrupt loose-packed cell aggregates. The rigid-packed cells were precipitated by gravity for 3 min and cell suspension was removed. The cell aggregates or cell spheres were then filled with 1 mL PBS and pipetted again. The procedure of collection, pipetting, precipitation, and suspension-removal was repeated for 3-times. The loose-packed cell aggregates were disrupted, while cell spheres remained intact. The remained cell spheres or cell aggregates were finally collected into microcapillary tube (VWR international, Radnor, PA, USA) and the bottom was stopped by toothstick connected with silicon-tube. The vertical-precipitated spheres were photographed and the lengths of cell columns were recorded, and the level of sphere formation was quantified [20].

### Side population analysis by flow cytometry

Parental or drug-selected melanoma cells were dissociated with trypsin/EDTA, and pelleted by centrifugation. The cells were resuspended at 1x10^6^ cells/mL in culture medium. Hoechst33342 dye was added to a final concentration of 5 μg/mL and cells were incubated at 37°C for 90 mins. Propidium iodide was then added to final concentration of 5 μg/mL to label dead cells. The cells were filtered through 80 μm mesh (BD bioscience, Inc., San Jose, CA, USA) to obtain a single cell suspension before analysis. Analysis was performed on a MoFlo XDP (Beckman Coulter Taiwan Inc., Taipei City, Taiwan). The Hoechst33342 dye was excited at 355 nm and its fluorescence was dual-wavelength analyzed with emission for Hoechst blue at 445 nm, and Hoechst red at 650 nm. Side population fractions were selected by the cells with low Hoechst blue and low Hoechst red intensities as confirmed by verapamil pretreatment. The percentage was determined by Kaluza software (Beckman Coulter Taiwan Inc., Taipei City, Taiwan).

### Xenograft animal experiments

The animal experiments was supervised and regulated by Experimental Animal Care and Use Committee in FuJen Catholic University with approval number A10452. The melanoma cells (parental cells, or drug-selected subpopulations) were inoculated subcutaneously at left or right side of nude mice (Nu/Nu strain in 3 weeks old, male) according the experimental designs. Basically, 2 x 10^3^ melanoma cells were inoculated, and the mice were supplemented with minimal food in specific pathogen free environment for tumor growth in 3 months. The tumor mass was recorded twice a week. The animals were sacrificed, and the tumor tissues were dissected and preserved in 10% (v/v) buffered formalin.

### Microarray experiments for gene expression profiling

Total RNA was extracted using Trizol reagent (Thermo Fisher Scientific Inc., Pittsburgh, PA, USA) and characterized to ensure good quality (OD_260_/OD_280_ >1.8) for array analysis. The complementary RNAs were amplified and labeled by TotalPrep RNA amplification kit (Illumina Inc., San Diego, California, USA). 1.5 μg of biotinylated cRNA will be used in hybridization onto HumanHT-12 v4 expression beadchip (Illumina Inc., San Diego, California, USA) for 16 hrs at hybridization chamber (58°C) according to manufacturer's procedure. The beadchip was washed and the hybridized probes were detected by Cy3-streptavidin using Illumina BeadArray Reader (Illumina Inc., San Diego, California, USA). Raw data of average probe signals were collected and the background for each bead was subtracted. The normalization data were further filtrated to keep statistically significant genes (*p* value of average probe signals ≥0.05). The fold change was defined as: (expression level in suspended melanoma cells / expression level in adherent melanoma cells). The array data was deposited in NCBI Gene Expression Omnibus (GEO) database (http://www.ncbi.nlm.nih.gov/geo/) with the accession number of GSE135689.

The genes with the value greater than 2.0 or less than 0.5 were considered for gene ontology classification by DAVID (Database for Annotation, Visualization and Integrated Discovery), and functional gene enrichment analysis by GSEA (gene set enrichment analysis). The changed genes were further analyzed by web-accessible program, DAVID (The Database for Annotation, Visualization and Integrated Discovery), to classify the functional annotation clustering of gene ontology in biological process, cellular component, and molecular function [58, 59]. Preranked gene set enrichment analysis (GSEA) and leading edge analysis [60, 61] were done to enrich gene sets associated with oncogenic signatures (C6 collection in MSigDB v4.0) with parameter of 1000 permaturation.

### Quantitative real-time PCR and statistical analysis

Total RNA was extracted and cDNAs were synthesized by MMLV HP reverse transcriptase (Epicentre, Madison, WI, USA) using freshly prepared RNA (1 μg) as PCR template. The samples were incubated as following procedure: 70°C to denature DNA for 10 min, 42°C to perform reverse transcription for 1 hr, and 95°C to inactivate reverse transcriptase for 5 min.

Quantitative real-time PCR and melting curve analysis were performed using VeriQuest Fast SYBR green qPCR reagent (Affymetrix Inc., Santa Clara, California, USA) in a StepOne Plus Real-time PCR system (Thermo Fisher Scientific Inc., Pittsburgh, PA, USA) using gene-specific primers. The qPCR reactions were carried out with the following parameters: one cycle at 95°C for 5 min; 40 cycles at 95°C for 30 sec, 60°C for 30 sec, 72°C for 30 sec; and a final elongation step at 72°C for 5 min. The 2^-ΔΔCT^ methods were used to determine the relative gene expression level by taking GAPDH as control gene. The data were expressed as mean ± standard deviation. Statistical analysis was done by Origin7.0 software (OriginLab Corporation, Northampton, Massachusetts, USA) using paired/one-tailed two sample t-test. The *p*-value of < 0.05 or < 0.01 was statistically significant and was indicated accordingly.

The forward and reverse primers used in this study were: NGFR, cctacggctactaccaggatg and cacacggtgttctgcttgtc; CD13, gccgtgtgcacaatcatcgcact and caccagggagcccttgaggtg; CD90, cccagtgaagatgcaggttt and gacagcctgagagggtcttg; CD24, tccaaggcacccagcatcctgctaga and tagaagacgtttcttggcctgagtct; Ki67, attgatcgttccttcaggtatg and tcatcagggtcagaagagaag; CD133, tctctatgtggtacagccg and tgatccgggttcttacctg; ALCAM, agtctt cattatcaggatgc and gggatcagttttctttgtca; c-Myc, gtcttcccctaccctctcaac and tccacagaaacaacatcgatttc; EpCAM,ctccacgtgctggtgtgt and tgttttagttcaatgatgatccagta; SOX2, cgagataaacatggcaatc and gccctttttaaacaagaccac; ANGPTL4,ccacttgggaccaggatcac and cggaagtactggccgttgag; NDST1, gggcgtggaggtttctaggaa and ctcctcctccttctcctccatcag; HS2ST1, tgaccctgtcttcacctttgtt and atgggcaaatccaactacagcagaa; SDC1, gctctggggatgactctgac and gtattctcccccgaggtttc; SDC2, ccagccgaagaggatacaaa and gcgttctccaaggtcatagc; SDC4, gtctggctctggagatctgg and tgggggctttcttgtagatg; GAPDH, gagtcaacggatttggtcgt and gatctcgctcctggaagatg;

### Phosphor-kinase antibody array experiment and Western blot analysis

The cell lysates were collected as followed. The adherent cells (60-80 % confluence) were washed twice by PBS, disrupted by lysis buffer (10 mM Tris-HCl, 5 mM EDTA, pH8.0, 1 % TritonX100, and protease inhibitors), and collected by a cell scraper. The suspended cells were collected and centrifuged. The cell pellet was washed by PBS twice and disrupted by lysis buffer. The lysate was kept on ice for 30 min and then centrifuged at maximum speed using a desktop centrifuge at 4°C for 10 min. Protein concentrations were quantified by a protein assay kit (Bio-Rad Laboratories Inc., Hercules, CA, USA).

For antibody array experiment, the activation level of different receptor tyrosine kinases were visualized by PathScan® RTK Signaling Antibody Array Kit #7982 (Cell signaling Inc., Danvers, Massachusetts, USA.) with chemiluminescent detection. The experimental procedure was according to vendor’s instruction. The expression of phospho- proteins were quantified by Image-J [62].

For western blot, protein lysate in 50 μg was subjected to SDS-PAGE and the separated proteins were transferred onto a PVDF membrane followed by 5% (w/v) skim-milk blocking. The membrane was then incubated in primary antibodies (1:1000 in 5% skim-milk in TBST) for 2 hrs at room temperature, and HRP-conjugated secondary antibody (1:10000) for 2 hrs at room temperature followed by enhance chemiluminescent detection (Merck Ltd., Taiwan, Taipei City, Taiwan).

The primary antibodies used in western blot were as followed. The anti-phospho-tyrosine antibody was purchased from Cell signaling Inc., USA. The primary antibody against ANGPTL4 and beta-actin were purchased from GeneTex Inc., Taiwan.

### *In vitro* tube formation assay

The 24-well plate was coated with 200 uL prechilled growth factor-depleted matrigel and left in cell incubator for gel solidification. For cell pretreatment with antibody, trypsinized cells were incubated with desired amount of ANGPTL4 antibody for 30 min before plating. For cell pretreatment with chemical inhibitors, drug-selected melanoma cells were pretreated with 50 mM sodium chlorate [63, 64] or 2 mM xylopyranoide [65] for 24 hrs before trypsinization. Melanoma cells (in 1x10^5^ cells) were then evenly seeded onto Matrigel surface. It was left undisturbed in cell incubator for 8 hours and recorded the images. The basis of number counting for in vitro tubes was to determine the number of lines of aligned cells connected between two nodes. Wider tube would compose of several lines of aligned cells, and the numbers of tubes will be counted accordingly.

### Flow cytometry analysis

Protein expression analyzed by flow cytometry was performed accordingly [66]. The 1 x 10^5^ cells were incubated with 1 μg anti-ANGPTL4 primary antibody overnight at 4°C, and 0.5 μg FITC-labeled secondary antibody at room temperature for 1 hr. The labeled cells were then analyzed by Gallios flow cytometer (Beckman Coulter Life Sciences, Indianapolis, Indiana, USA).

## Supplementary Material

Supplementary Figure 1
