# Supplementary material for: Drug-selected population in melanoma A2058 cells as melanoma stem-like cells retained angiogenic features – the potential roles of heparan-sulfate binding ANGPTL4 protein
Source: Aging (Albany NY). 2020 Nov 10;12(22):22700–18. doi: 10.18632/aging.103890 (PMC7746371; doi:10.18632/aging.103890)
Supplement: Supplementary Figure 1 [file aging-12-103890-s001..pdf]

SUPPLEMENTARY FIGURE

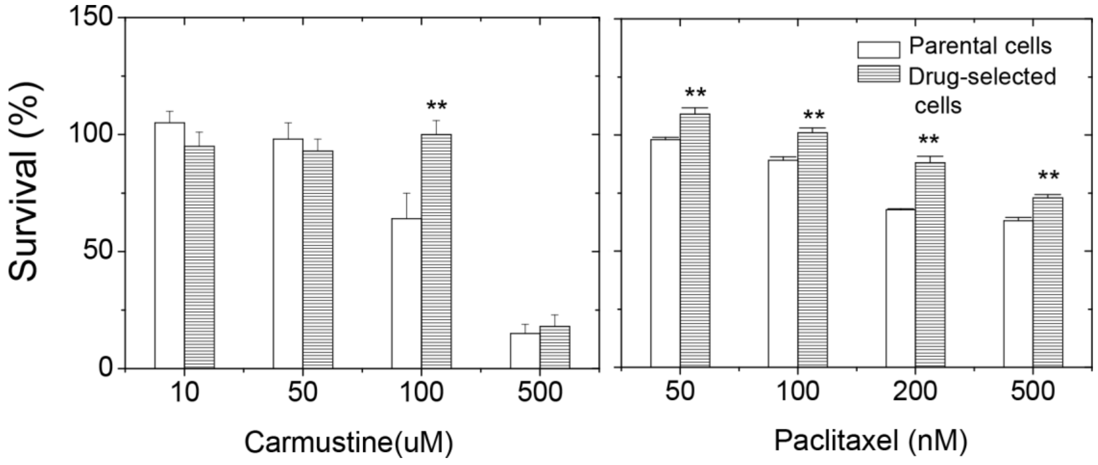

Supplementary Figure 1. Drug-selected cells were more resistant to the treatment of carmustine or paclitaxel.
